# Case Report: Kommerell's diverticulum and left aberrant subclavian artery stenosis hybrid treatment with branched aortic stent-graft

**DOI:** 10.3389/fcvm.2023.1309839

**Published:** 2023-12-12

**Authors:** Álvaro Rodríguez-Pérez, Cristina Tello-Diaz, A. Carolina Vergara-Budding, Constanza Fernández-Vinzenzi, Abdel Hakim Moustafa, Cesar Acebes Pinilla, Antonino Ginel-Iglesias, Antonio J. Barros-Membrilla, Jaime Felix Dilme-Muñoz

**Affiliations:** ^1^ Cardiology Department, Hospital de la Santa Creu I Sant Pau, UAB, IIB Sant Pau, CIBERCV ISCIII, Barcelona, Spain; ^2^ Department of Vascular and Endovascular Surgery, Hospital de la Santa Creu I Sant Pau, Institute of Biomedical Research (II-B Sant Pau), CIBER CV, UAB, Barcelona, Spain; ^3^ Cardiac Surgery Department, Hospital de la Santa Creu I Sant Pau, UAB, Barcelona, Spain; ^4^ Dimension Lab, Hospital de la Santa Creu I Sant Pau, UAB, Barcelona, Spain

**Keywords:** Kommerell's diverticulum, aberrant subclavian artery, subclavian steal syndrome, branched aortic stent-graft, right aortic arch, hybrid endovascular treatment

## Abstract

Kommerell's diverticulum in association with left or right aberrant subclavian arteries is a rare finding and is challenging to treat. Contemporary surgical and endovascular techniques provide a broad arsenal of possible treatments. Imaging techniques and modeling technology allow a more personalized strategy for each patient. In this case, we present a symptomatic patient with a Kommerell's diverticulum and a left aberrant subclavian artery complicated by proximal stenosis and poststenotic aneurysm. A hybrid technique using a single-branched thoracic stent-graft (Castor, MicroPort Medical, Shanghai, China) in combination with a surgical left subclavian-carotid bypass and endovascular occlusion of the poststenotic aneurysm using a vascular plug device (Amplatzer Vascular Plug, Abbott, Chicago, United States) was performed. This approach was planned and facilitated by the use of a 3D model. Alternative treatment options and the strengths of this approach are briefly reviewed and discussed.

## 1. Introduction

Kommerell's diverticulum is a rare vascular anomaly of the aortic branches, a persistent remnant of the fourth primitive dorsal arch, often associated with an aberrant subclavian artery. It can be present in both the left or right aortic arches, with aberrant right or left subclavian arteries, respectively (1). The prevalence of Kommerell's diverticulum and aberrant subclavian arteries is 0.4%–2.3% (2). Kommerell's diverticulum can be classified into three types according to the relationship with the subclavian artery; Kommerell's diverticulum in the left aortic arch with right aberrant subclavian artery (ASCA), Kommerell's diverticulum in the right aortic arch with left ASCA, and aortic diverticulum without ASCA (3).

The mean size of Kommerell's diverticulum at diagnosis ranges from 20 to 30 mm, with a mean growth rate of 1.5 mm/year in different series, and dissection or rupture have been widely described (3, 4). The clinical spectrum associated with this anomaly is wide and mainly depends on the trajectory and permeability of the aberrant subclavian artery; it can produce compressive symptoms including dysphagia, dyspnea, recurrent laryngeal nerve palsy, claudication, or even left subclavian steal syndrome if significant left subclavian artery stenosis is present. Other complications such as thrombosis and lower extremity emboli have also been described (3, 4).

Treatment indication has been suggested when the subclavian artery is aneurysmatic (>30 mm at the level of the diverticulum's orifice) and/or when the Kommerell's diverticulum measures more than 50 mm, measuring the cross-sectional area of the confluence with the descending aorta (1, 5). Treatment is also recommended in symptomatic patients regardless of size (2). Surgical, endovascular, and hybrid treatment approaches have been reported (5–9).

In this case, we present a symptomatic patient with a Kommerell's diverticulum and a left ASCA complicated by proximal stenosis and poststenotic aneurysm, treated using a hybrid technique. A novel single-branched thoracic stent-graft (Castor, MicroPort Medical, Shanghai, China) was used in combination with surgical left subclavian-carotid bypass and endovascular occlusion of the poststenotic aneurysm using a vascular plug device (Amplatzer Vascular Plug, Abbott, Chicago, United States). This approach allows less invasive treatment based on hybrid techniques and uses a commercial, non-customized graft that can be easily reproduced in other centers. Planning and monitoring were facilitated by using a 3D model obtained from an ECG-gated CT angiography.

## 2. Case presentation

A 41-year-old male patient with a past medical history of dyslipidemia and without other cardiovascular risk factors presented with a ten-year history of left arm positional claudication, without vertebrobasilar associated symptoms. There was no history of familiar cardiovascular disease.

Physical examination found symmetrical radial pulses, a lower blood pressure in the left arm (120/90 mmHg) compared to the right arm (140/95 mmHg), no heart or vascular murmurs, and a lack of neurological semiology.

A supra-aortic vessel Doppler ultrasound was performed. There was no atheromatosis and flow velocities were normal in the right vessels and left carotid artery. However, the left subclavian artery flow was blunted, with a proximal aliasing area up to 170 cm/s and a posterior blunted flow of 50 cm/s. In addition, the left vertebral artery flow was inverted, with a velocity of 48 cm/s, and was not modified by left arm hyperemia.

An ECG-gated CT angiography showed a right-sided aortic arch with normal diameters and four supra-aortic vessels, in the following order of origin: left common carotid artery, right common carotid artery, independent right subclavian artery, and aberrant left subclavian artery. The left subclavian artery origin was located in the descending thoracic aorta in relation to an aneurysmatic diverticulum in the left aortic wall, suggestive of a Kommerell's diverticulum with a maximum diameter of 30 mm. Immediately after the origin, the left subclavian artery had critical stenosis and a post-stenosis saccular aneurysm of 18 mm. The remaining left subclavian artery had a smaller diameter compared to the right subclavian artery, and the left vertebral artery was normal.

After these findings, a diagnosis of Kommerell's diverticulum in the right aortic arch with left aberrant subclavian artery and symptomatic critical stenosis and post-stenosis saccular aneurysm was made.

**Figure 1:**
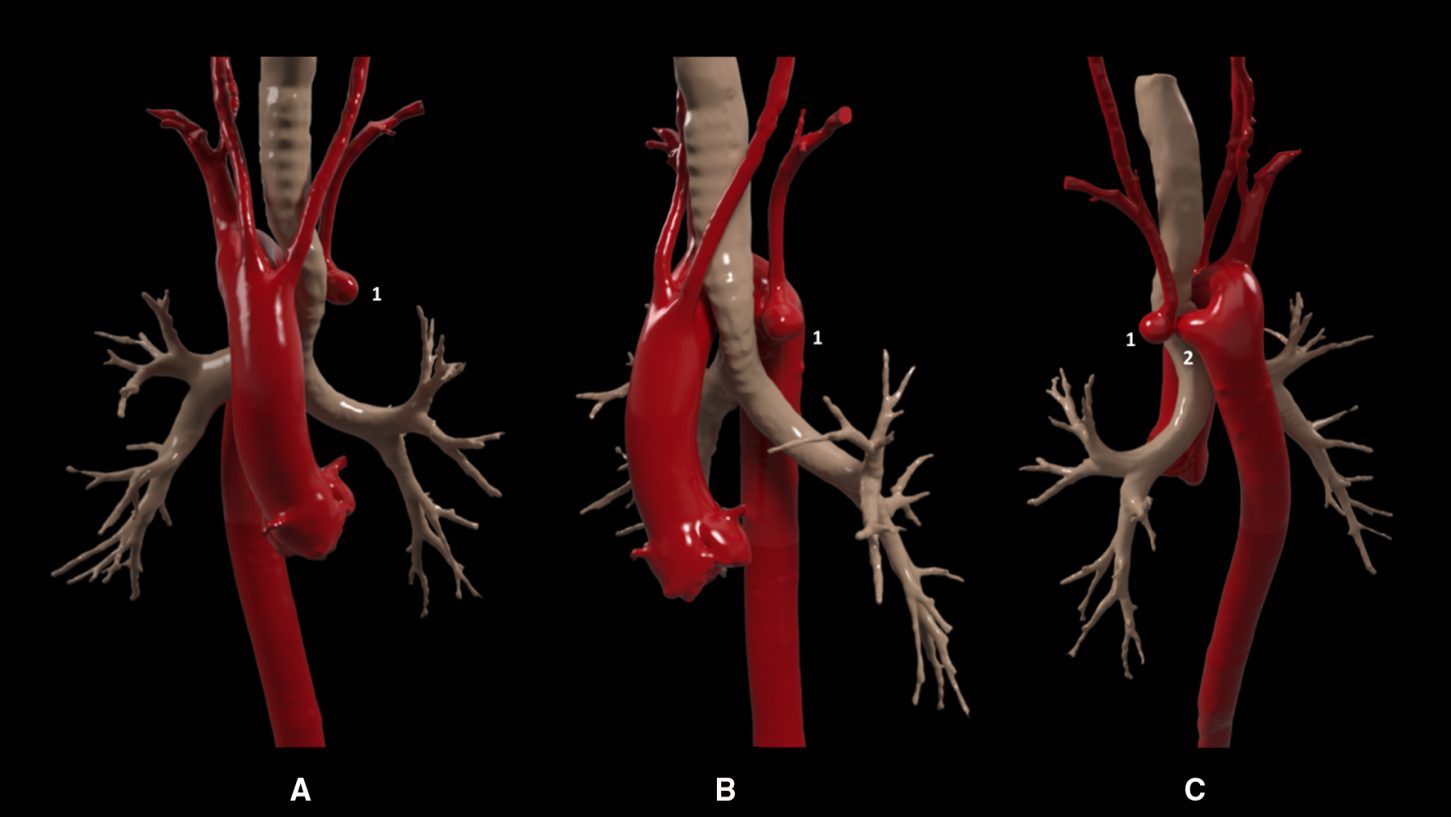
Pre-interventional 3D model. Anterior (**A**), lateral (**B**), and posterior (**C**) views showing a right-sided aortic arch and supra-aortic vessels, in the following order of origin: the left common carotid artery, right common carotid artery, independent right subclavian artery, and finally an aberrant left subclavian artery with critical proximal stenosis and post-stenosis saccular aneurysm (1) in relation to a Kommerell's diverticulum (2).

The treatment proposed by a multidisciplinary cardiovascular team was exclusion of the Kommerell's aneurysm and the post-stenotic saccular aneurysm to reduce the risk of rupture and treatment of the left subclavian artery critical stenosis to reduce the claudication symptoms. A 3D model of the aorta was obtained to better study the case (Figure 1). Normal ventricular and valvular function was found on preoperative transthoracic echocardiogram. Open surgery was evaluated as the first treatment option; however, after carefully studying the case, a surgical approach via left or right thoracotomy could guarantee proximal and distal control of the descending aorta. Considering the anatomical limitations and, therefore, the high surgical risk, a hybrid approach was preferred.

A left carotid-subclavian bypass followed by exclusion of the Kommerell's diverticulum using a one-branch thoracic endograft (Castor, MicroPort®) and exclusion of the post-stenotic saccular aneurysm using an Amplatzer Vascular Plug II was planned. The approach was explained to the patient and informed consent to treatment was obtained.

**Figure 2:**
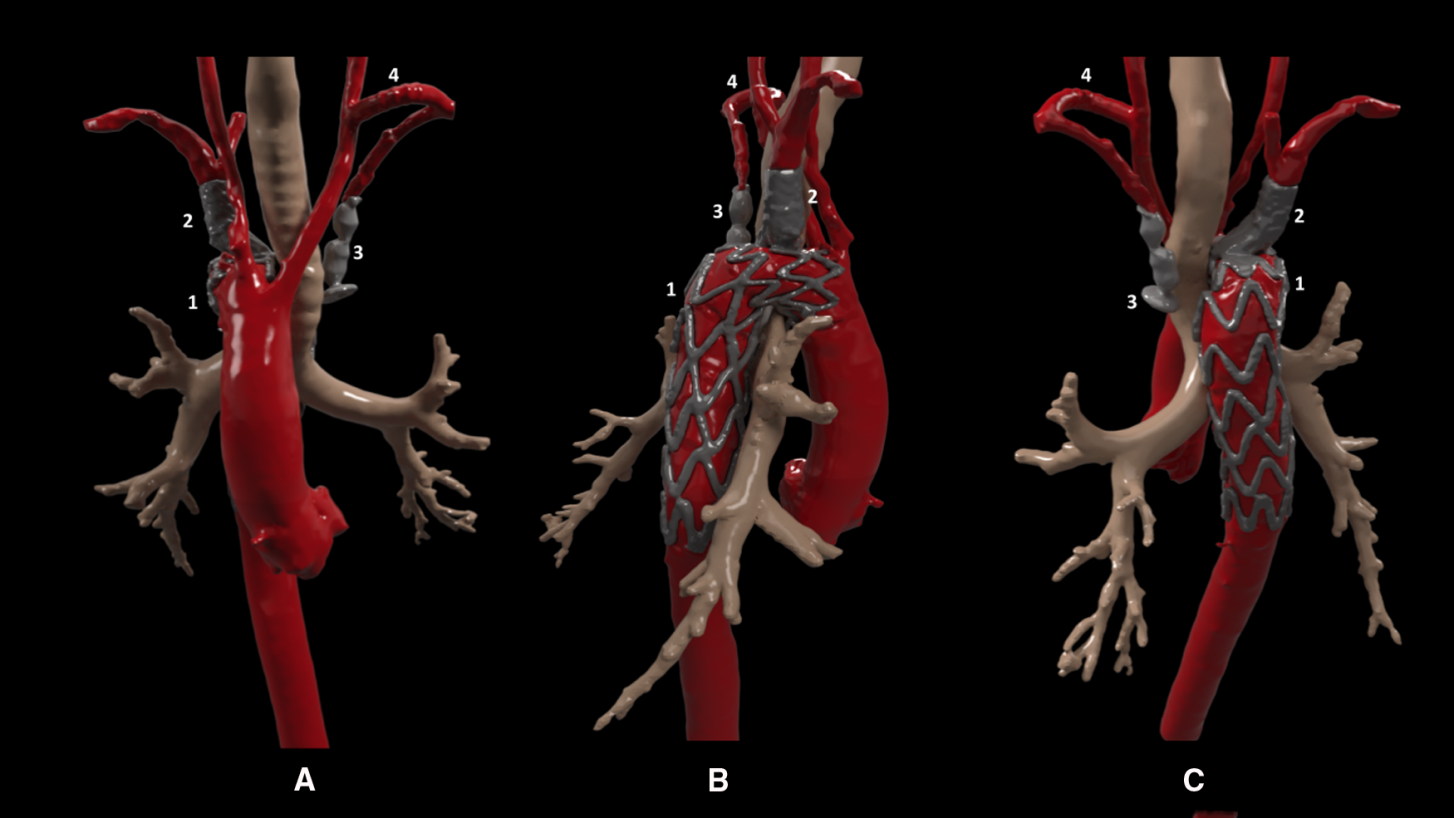
Post-interventional 3D model. Anterior (**A**), lateral (**B**), and posterior (**C**) views showing (1) the one-branch thoracic endograft (Castor, MicroPort®) in aorta with the branch placed in the right subclavian artery, extended with a covered stent, (2) Amplatzer Vascular Plug II, (3) in the left subclavian artery excluding Kommerell's diverticulum the poststenotic saccular aneurysm (therefore not visible in the model), and (4) left carotid-subclavian extra anatomical bypass.

Under general anesthesia, the left supraclavicular approach was used to perform a left carotid-subclavian bypass with a polytetrafluoroethylene (ePTFE) heparin-coated 6 mm ringed vascular prosthetic graft. The patient woke up without complications. One day after, the endovascular procedure was performed under general anesthesia. Open surgical access was performed for the right axillary artery and left humeral artery, and percutaneous access was performed for both femoral arteries. The through and through technique was used from the right femoral access to the right axillary access to facilitate the placement of the endograft and the deployment of the branch in the right subclavian artery. During deployment maneuvers, the endograft advanced slightly forward, partially occluding the ostium of the right common carotid artery. This was solved by performing distal traction of the endograft with a Reliant® balloon, managing to withdraw it a few millimeters. A lengthening of the coverage of the right subclavian artery was performed to avoid an excessive angulation using a 10 × 27 mm covered stent (iCover, iVascular, Barcelona, Spain). Finally, from the left humeral access, a 14 mm Amplatzer Vascular Plug II was placed, excluding the Kommerell's diverticulum and the saccular aneurysm of the left subclavian artery (Figure 2, intraoperative images provided in Supplementary Image S1–S4).

There were no immediate postoperative complications. Dual antiplatelet treatment was started with aspirin and clopidogrel and the patient was discharged on the fourth day of hospitalization.

**Figure 3:**
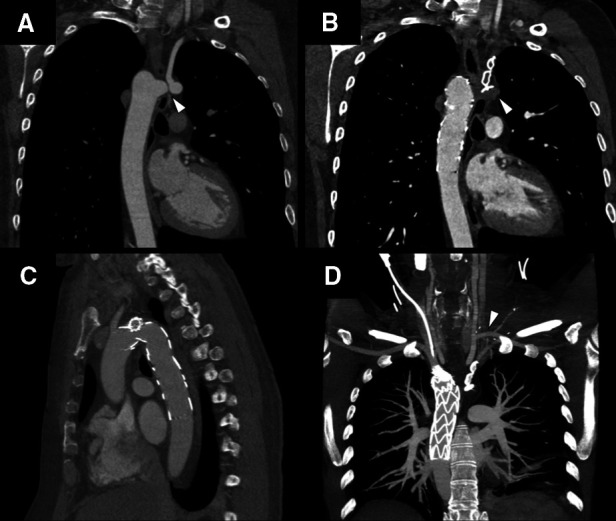
ECG-gated CT angiography panel. (**A**) Pre-interventional image, Kommerell's diverticulum and aberrant left subclavian with critical stenosis and poststenotic saccular aneurysm (white arrowhead). (**B**) Post-interventional image, complete exclusion and thrombosis of Kommerell's diverticulum and poststenotic aneurysm (white arrowhead). (**C**) Branched thoracic endograft (Castor, MicroPort®), correct alignment without endoleaks or thrombotic complications. (**D**) Maximum intensity projection (MIP) image showing supra-aortic vessel permeability. From left to right as seen in the image; right subclavian artery, right carotid artery, left carotid artery, extra anatomical bypass from the left carotid to left subclavian artery (white arrowhead), and native left subclavian artery excluded with an Amplatzer Vascular Plug II device.

The left arm claudication disappeared and a control ECG-gated CT angiography was performed 1 month after the intervention, which showed the complete thrombosis of the Kommerell's diverticulum and the post-stenotic aneurysm, the perfusion of all the supra-aortic branches was correct, and the endograft was well positioned without endoleaks or thrombotic complications (Figure 3). After 6 months of follow-up, the patient is still under dual antiplatelet therapy and free of left arm claudication symptoms.

## 3. Discussion

While Kommerell's diverticulum is a rare entity nowadays, incidental diagnosis of vascular anomalies is increasing due to the extensive use of a variety of cardiovascular imaging techniques. Treatment indication therefore requires a careful clinical and anatomical evaluation to determine if symptoms are caused by the anomaly and to determine the risk of complications. In this case, the correlation between claudication symptoms, the physical examination, and the ultrasound evaluation, along with the ECG-gated CT angiography information was clear and led to treatment indication besides the diverticulum's diameter.

As shown before, treatment indication for ASCA and Kommerell's diverticulum has been suggested in the American Heart Association (AHA) 2022 guidelines in the presence of symptoms related to the ASCA or a diameter of 30 mm at the level of the orifice of the diverticulum (1). According to the guidelines of the European Association for Cardio-Thoracic surgery (EACTS) and the European Society for Vascular Surgery (ESVS), the surgical approach for an ASCA and Kommerell's diverticulum should include the removal of the aberrant artery, with further subclavian-carotid transposition or bypass, the excision of the diverticulum, and proper aortic reconstruction with graft replacement of the descending aorta or total arch replacement (2, 6). Multiple surgical approaches have been described depending on the patient's anatomy and the presence of concomitant anomalies (7); however, there are still cases where the anatomy of the aortic arch and the descending aorta limits the surgical approach. In these cases, hybrid techniques combining surgical and endovascular approaches have been reported; the frozen elephant trunk technique in association with a TEVAR placement and coiling of the diverticulum with subclavian-carotid transposition or bypass and endovascular techniques with custom-designed endovascular grafts to exclude the ASCA and diverticulum have also been reported (8, 9). In reviewing the literature, less data is available regarding endovascular techniques with commercial branched endovascular grafts.

In this case, the hybrid approach of left carotid-subclavian bypass with exclusion of the diverticulum using a commercial one-branch thoracic endograft (Castor, MicroPort®) associated with exclusion of the post-stenotic saccular aneurysm using a vascular plug is an alternative exportable and efficient approach to treat these patients.

The approach reproducibility is limited, in any case, to each patient's anatomy, and requires a detailed evaluation and planification of every therapeutic alternative. In the case we describe, 3D modeling permitted a better understanding of the relations between structures and the *in silico* planification of the hybrid procedure, along with traditional CT reconstructions.

This situation is, therefore, a good example of the trend toward a more personalized medicine that we are experiencing nowadays, which requires modern technology and a high level of coordination and cooperation between the different areas involved in the treatment of these patients, such as cardiovascular imaging professionals and vascular and cardiac surgeons.

## Data Availability

The original contributions presented in the study are included in the article/**Supplementary Material**, further inquiries can be directed to the corresponding author.

## Supplementary material

Supplementary Image S1 Intraoperative image showing a sharp angulated aortic arch with the folded branched thoracic endograft and the right subclavian branch deployed.

Supplementary Image S2 Intraoperative image comparing the endograft position before (**A**) and after (**B**) performing a corrective distal traction to avoid partial occlusion of the right common carotid artery. The endotracheal tube (red line) is used as a reference.

Supplementary Image S3 Intraoperative image after complete deployment of the endograft.

Supplementary Image S4 Intraoperative images showing the extension of the endograft branch with a covered stent to avoid excessive angulation in the right subclavian artery. Initial incorrect apposition of the thoracic endograft branch in the right subclavian artery with excessive angulation (**A**). Coverage with a stent to the origin of the dominant right vertebral artery (**B**). Final image of the realigned endograft branch after covered stent implantation (**C**).

## References

[1] Isselbacher EM Preventza O Black JH Augoustides JG Beck AW Bolen MA 2022 ACC/AHA guideline for the diagnosis and management of aortic disease: a report of the American heart association/American college of cardiology joint committee on clinical practice guidelines. Circulation. (2022) 146:334–482. 10.1161/CIR.0000000000001106 PMC9876736 36322642

[2] Czerny M Schmidli J Adler S van den Berg JC Bertoglio L Carrel T Current options and recommendations for the treatment of thoracic aortic pathologies involving the aortic arch: an expert consensus document of the European association for cardio-thoracic surgery (EACTS) & the European society for vascular surgery (ESVS). Eur J Vasc Endovasc. (2019) 57:165–98. 10.1016/j.ejvs.2018.09.016 30318395

[3] Erben Y Brownstein AJ Velasquez CA Li Y Rizzo JA Mojibian H Natural history and management of kommerell’s diverticulum in a single tertiary referral center. J Vasc Surg. (2020) 71:2004–11. 10.1016/j.jvs.2019.08.260 31708305

[4] van Rosendael PJ Stöger JL Kiès P Vliegen HW Hazekamp MG Koolbergen DR The clinical spectrum of Kommerell’s diverticulum in adults with a right-sided aortic arch: a case series and literature overview. J Cardiovasc Dev Dis. (2021) 8:1–17. 10.3390/jcdd8030025 PMC7996811 33652796

[5] Noda M Ishikawa H Takami Y Sakurai Y Amano K Akita K Hybrid repair for Kommerell’s diverticulum and right aortic arch with aberrant right vertebral artery. Fujita Med J. (2022) 8:34–6. 10.20407/fmj.2020-016 35233346 PMC8874913

[6] Tanaka A Milner R Ota T. Kommerell’s diverticulum in the current era: a comprehensive review. Gen Thorac Cardiovasc Surg. (2015) 63:245–59. 10.1007/s11748-015-0521-3 25636900

[7] Karangelis D Loggos S Tzifa A Mitropoulos FA. The aberrant subclavian artery: approach to management. Curr Opin Cardiol. (2020) 35:636–42. 10.1097/HCO.0000000000000793 32852349

[8] Gafoor S Stelter W Bertog S Sievert H. Fully percutaneous treatment of an aberrant right subclavian artery and thoracic aortic aneurysm. Vasc Med. (2013) 18:139–44. 10.1177/1358863X13485985 23720037

[9] Silveira P Narciso R Ferreira JR Torres T Galego G Torres C. Total endovascular repair of aberrant left subclavian artery with Kommerell’s diverticulum using a customized branched device. J Vasc Surg. (2013) 57:1123–5. 10.1016/j.jvs.2012.10.008 23312832

