# Supplementary figures and images for: Case Report: Kommerell's diverticulum and left aberrant subclavian artery stenosis hybrid treatment with branched aortic stent-graft
Source: Front Cardiovasc Med. 2023 Dec 12;10:1309839. doi: 10.3389/fcvm.2023.1309839 (PMC10754508; doi:10.3389/fcvm.2023.1309839)

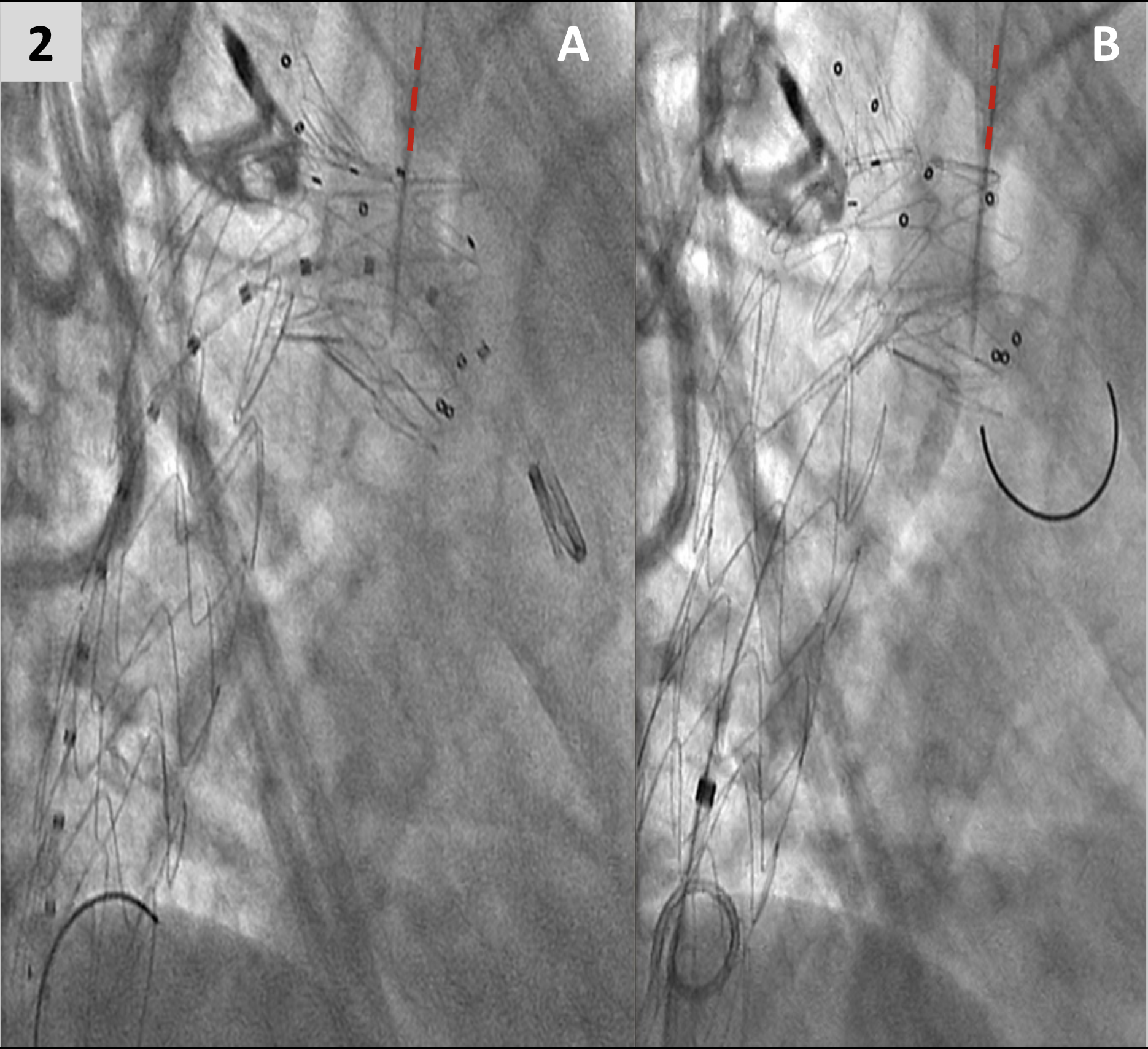

Supplement: Supplementary Image S2 — Intraoperative image comparing the endograft position before (A) and after (B) performing a corrective distal traction to avoid partial occlusion of the right common carotid artery. The endotracheal tube (red line) is used as a reference. [file Image2.tiff]

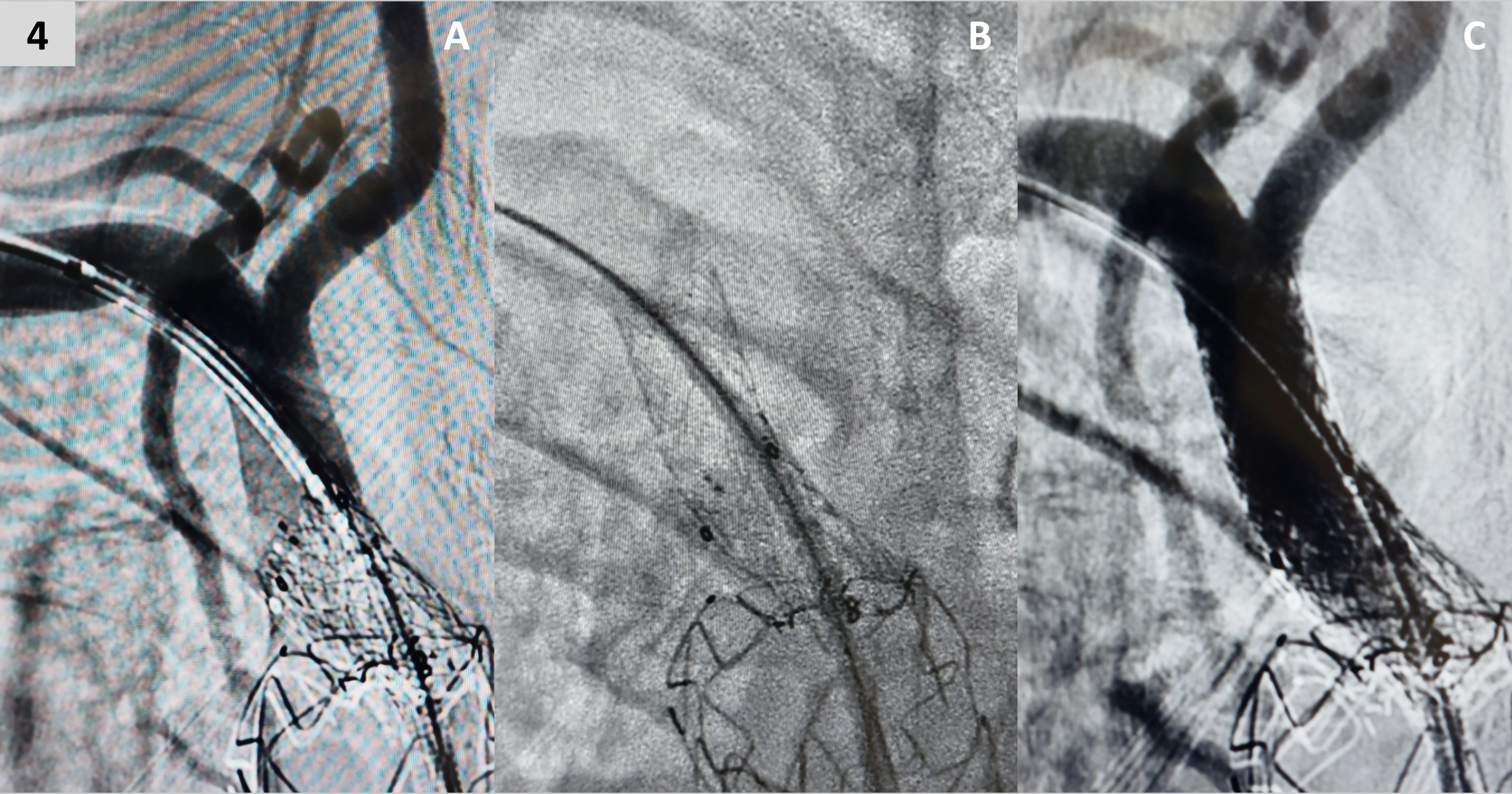

Supplement: Supplementary Image S4 — Intraoperative images showing the extension of the endograft branch with a covered stent to avoid excessive angulation in the right subclavian artery. Initial incorrect apposition of the thoracic endograft branch in the right subclavian artery with excessive angulation (A). Coverage with a stent to the origin of the dominant right vertebral artery (B). Final image of the realigned endograft branch after covered stent implantation (C). [file Image4.tiff]
